# Case report: Late-onset MELAS syndrome with mtDNA 5783G>A mutation diagnosed by urinary sediment genetic testing

**DOI:** 10.3389/fgene.2024.1367716

**Published:** 2024-05-31

**Authors:** Hao Cai, Li-Min Li, Miao Zhang, Yuying Zhou, Pan Li

**Affiliations:** ^1^ Department of Neurology, Tianjin Huanhu Hospital, Tianjin, China; ^2^ Tianjin Key Laboratory of Cerebral Vascular and Neurodegenerative Diseases, Tianjin Neurosurgery Institute, Tianjin Huanhu Hospital, Tianjin, China; ^3^ Clinical College of Neurology, Neurosurgery and Neurorehabilitation, Tianjin Medical University, Tianjin, China; ^4^ Tianjin Huanhu Hospital Affiliated to Tianjin University Huanhu Hospital, Tianjin, China; ^5^ Department of Neurology and Tianjin Neurological Institute, Tianjin Medical University General Hospital, Tianjin, China

**Keywords:** MELAS, late-onset, MT-TC, m.5783G>A, urinary sediment

## Abstract

**Background:**

Patients with mitochondrial encephalomyopathy, lactic acidosis, and stroke-like episodes (MELAS) usually present with multisystemic dysfunction with a wide range of clinical manifestations. When the tests for common mitochondrial DNA (mtDNA) point mutations are negative and the mtDNA defects hypothesis remains, urine epithelial cells can be used to screen the mitochondrial genome for unknown mutations to confirm the diagnosis.

**Case presentation:**

A 66-year-old Chinese woman presented with symptoms of MELAS and was initially misdiagnosed with acute encephalitis at another institution. Although genetic analysis of blood lymphocyte DNA was negative, brain imaging, including magnetic resonance imaging, magnetic resonance spectroscopy, and clinical and laboratory findings, were all suggestive of MELAS. Finally, the patient was eventually diagnosed with MELAS with the mtDNA 5783G>A mutation in the MT-TC gene with a urinary sediment genetic test.

**Conclusion:**

This case report expands the genetic repertoire associated with MELAS syndrome and highlights the importance that full mtDNA sequencing should be warranted beside the analysis of classical variants when a mitochondrial disorder is highly suspected. Furthermore, urine sediment genetic testing has played a crucial role in the diagnosis of MELAS.

## Introduction

Mitochondrial encephalomyopathy, lactic acidosis and stroke-like episodes (MELAS) is a matrilineal inherited multisystem disease characterized by stroke-like episodes accompanied by seizures, headache, hemiparesis, cortical blindness, hearing disability, and diabetes mellitus (Pavlakis et al., 1984; Goto et al., 1992). The main underlying mechanism of the disease is caused by mutations in mitochondrial DNA (mtDNA). Impairment of mitochondrial translation, which leads to a decline in protein synthesis and energy depletion, eventually results in mitochondrial dysfunction and an inability to generate adequate energy to support various organs (Koga et al., 2005; Alston et al., 2017). Approximately 80% of patients with MELAS have been reported to carry the A3243G mutation in the mitochondrial tRNA (leucine)-1 (MT-TL1) gene encoding tRNA leucine. The T3271C mutation is responsible for MELAS in approximately 7.5% of patients, but in up to 10% of patients with MELAS, the mtDNA mutations remain unclear (Goto et al., 1990; Goto, 1995; Lorenzoni et al., 2015). Although the mutations were initially found in DNA isolated from muscle, they are typically present in all tissues of patients and are less abundant in tissues of oligosymptomatic or asymptomatic maternal relatives (Shanske et al., 2004). Therefore, next-generation DNA sequencing from patients’ blood samples has become the first-line diagnostic tool (Baker et al., 2018). Some maternal relatives who were expected to be carriers by pedigree analysis also showed no detectable A3243G mutation in their blood. Thus, blood may not be the tissue of choice for the detection of carriers or for diagnosing MELAS. Recent studies have shown that urinary sediment seems to be a better choice for diagnosing mtDNA mutations, as it is readily available, and the mutation load is almost always greater than that in blood (Frederiksen et al., 2006; Ma et al., 2009; Marotta et al., 2009). Although the clinical symptoms of patients with MELAS typically appear before the age of 40, there is still a lack of comprehensive and unified diagnostic criteria (Leonard and Schapira, 2000). Here, the present case report described a 66-year-old female patient with MELAS harbouring the m.5783G > A mutation in the mitochondrial cysteine transfer RNA (MT-TC) gene who was diagnosed by a urinary sediment genetic test.

### Clinical case

**FIGURE 1:**
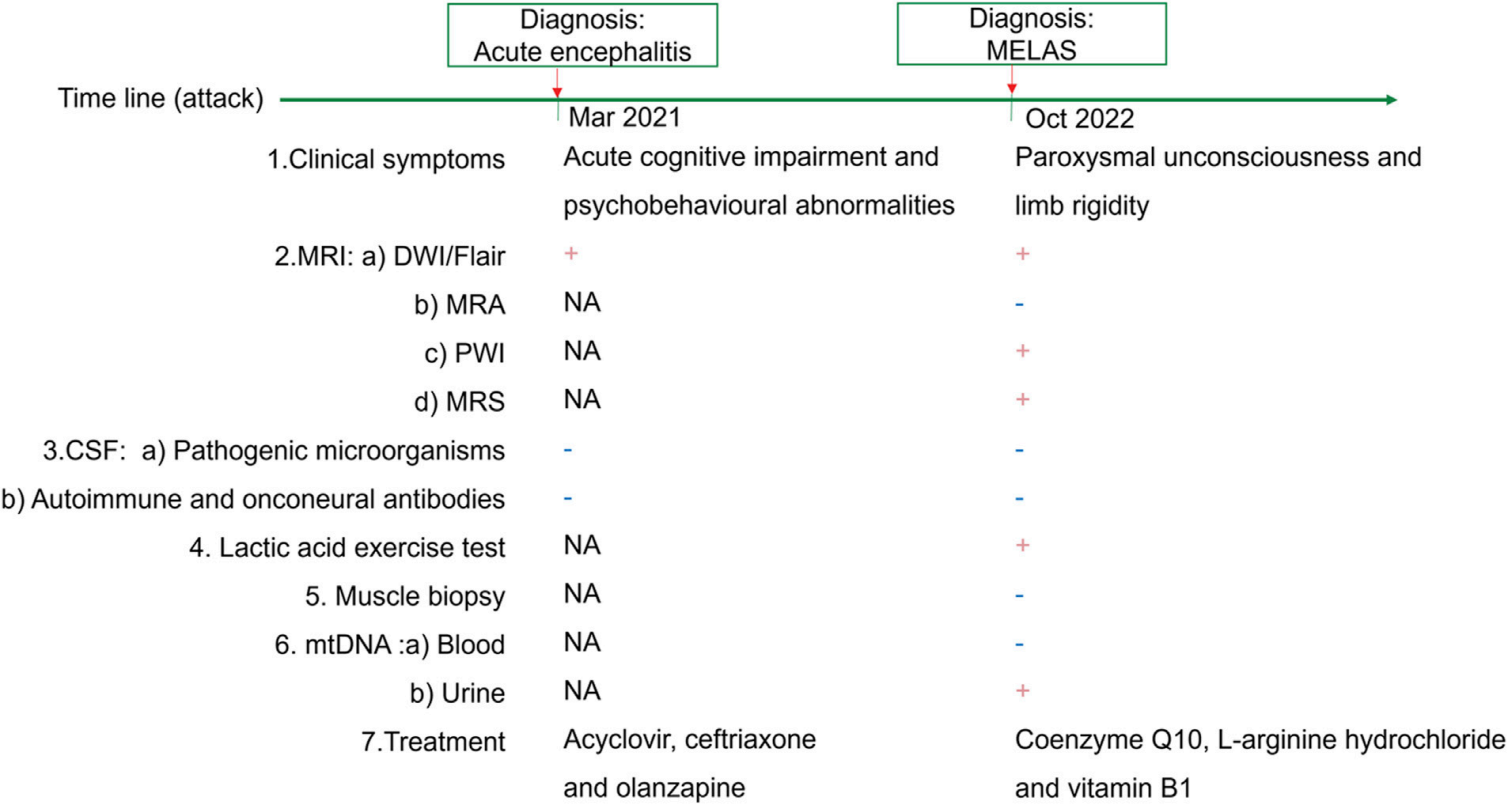
Timeline of MELAS and therapeutic regimens.

A 66-year-old female patient was first admitted to another hospital 1 day after the onset of acute cognitive impairment and psychobehavioural abnormalities, accompanied by hallucinations and fever (Figure 1). Her medical history included hypertension and myocardial ischaemia. She had a history of headache for many years, and unexplained vision loss occurred in the left eye more than 30 years prior. Her father was of short stature and diagnosed with dementia, and her mother suffered from heart failure. Her brother died of an unspecified illness 3 years prior. The patient had a daughter of slight stature.

**FIGURE 2:**
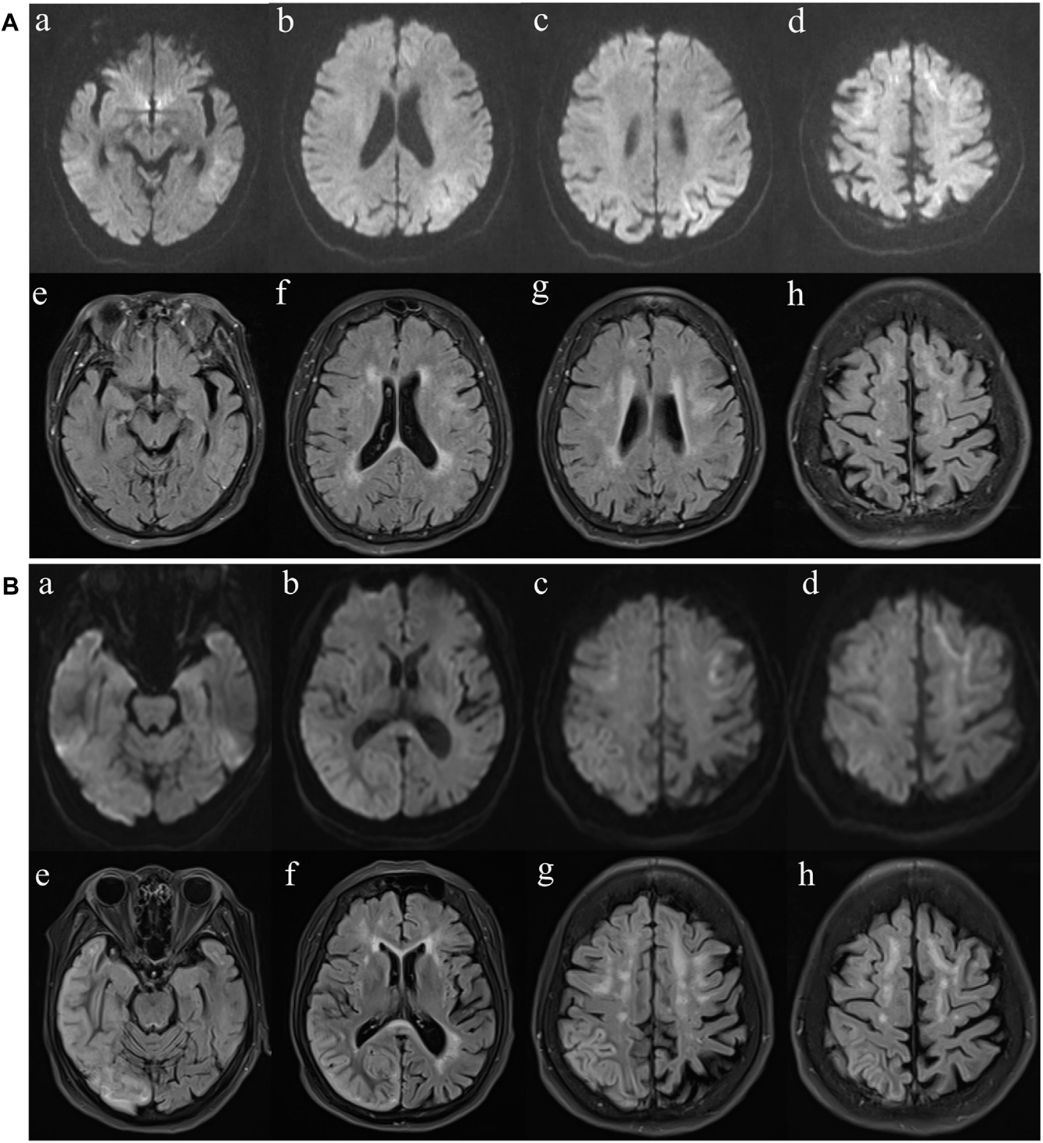
MR imaging findings. **(A)** Diffusion weighted imaging (DWI) (a-d) and fluid-attenuated inversion recovery (FLAIR) (e-h) images at first presentation. FLAIR imaging reveals high-intensity lesions in the bilateral frontal lobe and paraventricular area. The left temporal, parietal, occipital and bilateral frontal lobes are hyperintense on DWI. **(B)** Diffusion weighted imaging (DWI) (a-d) and fluid-attenuated inversion recovery (FLAIR) (e-h) images of the case at second presentation. FLAIR reveals high-intensity lesions in cortical and subcortical areas of the right frontal, temporal, parietal and occipital lobes. The bilateral frontal lobes and corpus callosum are hyperintense on DWI.

Upon admission, she was 148 cm tall, and she weighed 43 kg. On physical examination, her body temperature was 37.5°C. No significant positive signs were found in the heart, lung or abdomen. Neurological examination showed mild disturbance of consciousness: GCS 12 (E3 V4 M5), attention disorder, disorientation, psychosis manifesting as verbal abuse, abnormal behaviours and hallucinations. Brain magnetic resonance imaging (MRI) revealed high-intensity lesions in the left temporoparietal and occipital areas and bilateral frontal lobe area on diffusion weighted imaging (DWI) and fluid-attenuated inversion recovery (FLAIR) imaging (Figure 2A). The patient underwent a lumbar puncture, and cerebrospinal fluid (CSF) examination revealed that the pressure was 162 mmH_2_O. Pandy’s test was weakly positive. The total cell count in the CSF was 96 × 10^6/L, and the white blood cell count was 8 × 10^6/L. CSF biochemistry showed that the protein level was 0.71 g/L, glucose was 5.20 mmol/L and lactic acid was 2.8 mmol/L. Electroencephalography (EEG) showed high-amplitude, irregular slow waves and no definite epileptic wave emission. The patient was initially diagnosed with acute encephalitis with an infectious or autoimmune origin and treated with acyclovir and ceftriaxone. Olanzapine was also administered to control psychotic symptoms. Then, the detection of pathogenic microorganisms in the CSF was negative. Moreover, autoimmune encephalitis-associated antibodies and onconeural antibodies were all negative. Enhanced MRI of the brain did not show significant enhancement, and the abnormal signal range on DWI was reduced. In view of the improvement in the patient’s state of consciousness and psychiatric symptoms after treatment, the patient’s family refused further examination and finally demanded automatic discharge.

**FIGURE 3:**
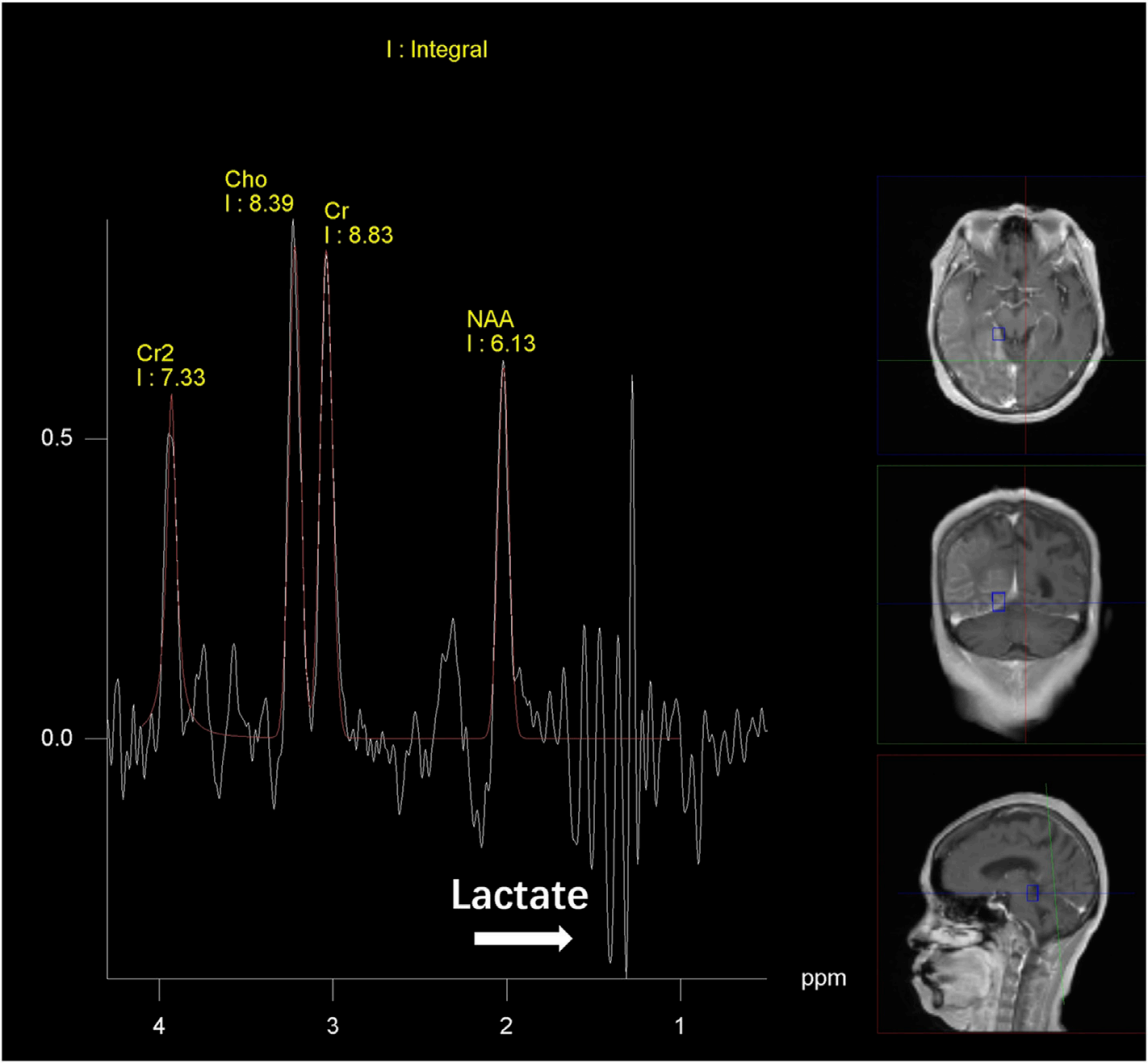
MR spectroscopy findings. Magnetic resonance spectroscopy (MRS) reveals a decreased N-acetyl aspartate (NAA) level and increased Cho and Cr levels and lactic acid peak in right frontal, temporal, parietal and occipital lesions relative to the contralateral, normal areas.

**FIGURE 4:**
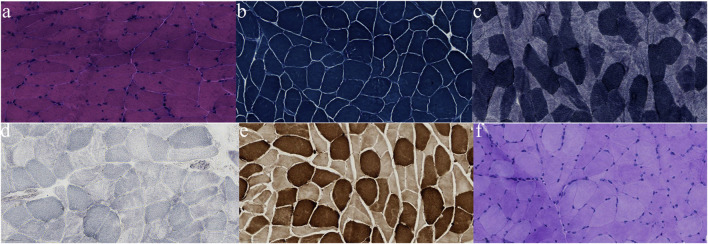
Pathological findings from biopsied muscle. No RRF was found on modified Gomori trichrome (MGT) staining **(A)**; no obvious disordered arrangement of myofibrillar reticulum was observed on nicotinamide adenine dinucleotide (NADH) staining **(B)**; no ragged-blue fibres or succinate dehydrogenase (SDH) strongly reactive blood vessels were observed on SDH staining **(C)**; no COX-negative fibres were found **(D)**; no significant increase in fat droplets was observed on Oil Red O (ORO) staining **(E)**; no significant increase in glycogen was detected with Periodic acid-Schiff (PAS) staining **(F)**.

After discharge, the patient continued to present with psychiatric symptoms and cognitive impairment but did not seek treatment again. One year later, she was admitted to our hospital due to paroxysmal unconsciousness with limb rigidity (Figure 1). A brain MRI obtained on admission showed an increased DWI/FLAIR signal in both frontal lobes and the corpus callosum. The right frontal, temporal, parietal, and occipital cortices showed oedema with a slightly elevated FLAIR signal (Figure 2B). Magnetic resonance angiography (MRA) did not show significant vascular stenosis or vascular occlusion. Brain MRI perfusion imaging was performed, and the results showed a decrease in time to peak (TTP) and mean transit time (MTT) and an increase in cerebral blood volume (CBV) and cerebral blood flow (CBF) in the right frontal, temporal, parietal, and occipital areas, which were considered hyperperfusion manifestations, while hypoperfusion manifestations in the left lateral ventricle were considered a compensatory phase. Magnetic resonance spectroscopy (MRS) revealed an elevated and inverted lactate peak with a decreased N-acetyl-aspartate level (Figure 3). Repeated CSF analyses clarified the elevated level of lactate. The patient underwent a lactic acid exercise test, and the results were positive. These ancillary test results contributed to a possible diagnosis of MELAS. With the diagnosis of suspected MELAS, the patient was treated with oral coenzyme Q10 (90 mg/day), L-arginine hydrochloride (7.5 g/day), and vitamin B1 (225 mg/day). For further histopathological diagnosis, muscle biopsy of the left biceps brachii of the patient was performed. However, the muscle biopsy results did not reveal ragged-red fibres (RRFs), which are the classic manifestation of MELAS (Figure 4). Genetic analysis (Guangzhou V-Medical Laboratory Co.) of blood lymphocyte DNA was negative for MELAS pathogenic variants. However, the urinary sediment genetic test (Guangzhou V-Medical Laboratory Co.) by next-generation sequencing (NGS) revealed a mtDNA 5783G → A point mutation (heteroplasmy level of the mutation was 94.15%), confirming the diagnosis of MELAS. With continued treatment, the patient’s disturbed consciousness and limb rigidity gradually improved, but her cognitive function remained impaired. On day 16, she was transferred to a rehabilitation hospital.

### mtDNA 5783G>A mutation analysis

The first morning urine sample of 30–40 mL was collected by a clean 50 mL centrifuge tube and centrifuged at 1,000 rpm for 10 min to obtain sediment. DNA was extracted using a commercially available DNA isolation kit (Hipure Tissue and Blood DNA Kit, Magen, China). Guangzhou V-Medical Laboratory provided the measurements of the urinary sediment genetic test using the NGS. The main detection reagents involved in this test were the KMM-101/KOD one TM PCR Master Mix (Toyobo, Japan) and DNA Library Fast Construction Kit (CWBIO CW3045M, China). The detection instrument was Nova 6000 (Illumina, United States).

## Discussion

The defining clinical features of MELAS syndrome include stroke-like episodes, seizures, visual disturbances, motor weakness, and headache. Additionally, hearing impairment, cortical blindness, and diabetes mellitus have been frequently described in adults (Pavlakis et al., 1984). Typical MELAS syndrome presents in childhood, with the majority of patients presenting before 40 years of age (Leonard and Schapira, 2000). Here, we report a case of a late-onset patient who initially presented with psychiatric symptoms and cognitive impairment. In the auxiliary examination, brain MRI showed abnormal signals in the bilateral frontal lobes, and lumbar puncture showed increased white blood cell count and protein levels, which indicated acute encephalitis possibly caused by infective or autoimmune factors. Negative results for pathogenic microorganisms, antibodies of autoimmune encephalitis and paraneoplastic syndrome suggested a further differential diagnosis, including MELAS. The clinical and neuroimaging findings prompted the analysis of mtDNA thanks to availability of urinary sediment DNA. The patient was eventually diagnosed with MELAS, and the presence of a 5783G>A point mutation in mtDNA was discovered with the assistance of urinary sediment genetic test results. No such mutation was detected in her daughter’s blood. Unfortunately, we did not test the urine of the patient’s daughter for mitochondrial mutations because her daughter refused our request. The follow-up studies should be conducted for the patient and her family.

MELAS syndrome is a genetically determined disease caused by mutations in mtDNA. The mtDNA A-to-G transition at nucleotide 3243 is the most common mutation (Goto et al., 1990; Kaufmann et al., 2011), however, this site mutation was excluded from urine and blood during the genetic screening of the patient. It is estimated that up to 10% of MELAS patients have undetected mtDNA mutations (Lorenzoni et al., 2015). In the present case, the most common mutation was not detected. However, MELAS syndrome was diagnosed by detection of a 5783G>A mutation in the MT-TC gene in the urinary epithelia. The mitochondrial MT-TC mutation may have pathogenic significance, as Kawazoe et al. reported a 68-year-old woman who presented with myoclonic epilepsy with RRF harbouring a novel homoplasmic m.5820C>A variant in the MT-TC gene (Kawazoe et al., 2022). Although the 5783G>A mutation is present in ClinVar and has been interpreted as Clinically benign (https://www.ncbi.nlm.nih.gov/clinvar/variation/689987/), it has also been confirmed the pathogenicity of the mutation (Feigenbaum et al., 2006). Feigenbaum et al. reported an 8-year-old girl with MELAS who presented with RRF myopathy, short stature, and deafness. Whole mitochondrial genome sequencing analysis was performed, and novel changes, including 5783G>A in tRNA^cys^, were found. The 5783G>A mutation occurs in the T arm of tRNA^cys^, leading to the disruption of the stem structure and possibly reducing the stability of the tRNA (16). The finding of 5783G>A in the structurally important T-arm stem region of tRNA^cys^ was added to the understanding of tRNA gene mutations. Meanwhile, several findings support the deleterious effects of the 5783G>A mutation. First, the mutation is located in the structurally/functionally important stem region of the T arm of tRNA^cys^. Second, the presence of this mutation in the tRNA gene is consistent with mitochondrial proliferation and mtDNA amplification. Third, significantly reduced respiratory chain activity was observed in all four complexes in muscle biopsies, consistent with widespread mitochondrial dysfunction due to impaired tRNA function. All this evidence strongly supports that this mutation is pathogenic (McFarland et al., 2002; Gropman et al., 2004; Tarnopolsky et al., 2004; Feigenbaum et al., 2006). In the case reported by Feigenbaum et al., patient with 5783G>A mutation had a younger onset age and a more severe condition, who ultimately developed renal failure and fatal cardiac dysfunction (Feigenbaum et al., 2006). Previous studies revealed a correlation between the proportion of mutant mtDNA in the age of onset and the affected tissues (Macmillan et al., 1993; Mariotti et al., 1995) and also the severity of the disease (Mariotti et al., 1995). While, Yokota et al. found that heteroplasmy at the single-cell level was widely varied among the primary fibroblasts derived from MELAS patients (Yokota et al., 2015), which suggests that the mean heteroplasmy level in the affected organ may not represent the disease burden. Further investigation is required to perform why this case showed later onset of the syndrome and an overall milder clinical course than that of the case reported by Feigenbaum et al. In a study on deafness, Meng et al. investigated the molecular mechanism of deafness-associated 5783C>T mutations that affect typical C50-G63 base repair in tRNA^Cys^ TC stems. The 5783C>T mutation alters the structure and function of tRNA^Cys^, including reducing the melting point and producing conformational changes, instability and defects in aminoacylation. Abnormal tRNA metabolism impairs mitochondrial translation, especially in polypeptides with higher numbers of cysteine and tyrosine codons. Then, insufficient oxidative phosphorylation, including instability and reduced activity of respiratory chain enzyme complexes I, III, and IV and intact supercomplexes, is ultimately present as a result of these alterations (Meng et al., 2022). The pathogenic mechanism underlying the 5783G>A mutation in the MT-TC gene may be similar, and studies on the mechanism of the mutation need to be further explored in the future.

In the present case, although the whole blood genetic test by NGS was negative, the clinical manifestations, MRI/MRS and laboratory test results of the patient all indicated that MELAS could not be excluded. Therefore, a urinary sediment genetic test was performed, and a disease-associated gene mutation was found. The levels of mutated mitochondrial genes may be low in the blood of probands, and even lower in asymptomatic or oligosymptomatic maternal relatives. This is because changes in the level of mtDNA heterogeneity are fundamentally related to the pathophysiology and clinical progression of mitochondrial diseases. Studies have confirmed that the percentage of mtDNA mutations in the blood decreases exponentially with age (Rajasimha et al., 2008). The mtDNA mutation rate is significantly different in different tissues, especially in adults, muscle tissue, urinary sediment cells and hair follicles have higher positive rates than that in peripheral blood cells (Ma et al., 2009). Recently, applications of single-cell genomics have identified a high prevalence of somatic mtDNA mutations, many of which are stably propagated, facilitating clonal/lineage tracing studies and possibly helping to improve the detection rate of pathogenic mtDNA variants associated with MELAS in the blood (Walker et al., 2020; Lareau et al., 2021). Nevertheless, Shanske et al. assessed the mutational loads of mitochondrial mutant genomes in other accessible tissues and found that the proportion of DNA mutated genomes was generally highest in urine sediment and lowest in blood (Shanske et al., 2004). Similarly, Marotta et al. reported a patient with a known family history of MELAS caused by the MT-TL1 m.3243A>G mutation, which was detected only in urine but not in hair, blood, or skeletal muscle (Marotta et al., 2009). In the research of Ma et al., the A3243G mutation rate in urine was significantly higher than that in blood in MELAS patients and carriers with minor symptoms or normal phenotypes (Ma et al., 2009). These findings suggest that assessment of the mtDNA mutation rate in urine is a noninvasive, convenient and rapid method with diagnostic significance superior to blood detection.

In conclusion, the case with late-onset MELAS with a 5783G>A mtDNA mutation where the clinical onset was masqueraded as acute encephalitis with an infective or autoimmune cause expands the genetic repertoire associated with MELAS syndrome. The case also highlights the importance that full mtDNA sequencing should be warranted beside the analysis of classical variants when mitochondrial disease is highly suspected (Feigenbaum et al., 2006). In addition, the analysis of urinary sediment testing emerged as pivotal in confirming the suspected diagnosis of MELAS, aiding in treatment adjustment, and improving the patient’s phenotype.

## Data Availability

The datasets for this article are not publicly available due to concerns regarding participant/patient anonymity. Requests to access the datasets should be directed to the corresponding author.
